# Exploring the Subclinical Atherosclerotic Load in Patients With Rheumatoid Arthritis: A Cross-Sectional Study

**DOI:** 10.7759/cureus.32644

**Published:** 2022-12-17

**Authors:** Arpit Arora, Vaibhav Ingle, Rajnish Joshi, Rajesh Malik, Gaurav Khandelwal

**Affiliations:** 1 General Medicine, All India Institute of Medical Sciences, Bhopal, IND; 2 Internal Medicine, All India Institute of Medical Sciences, Bhopal, IND; 3 Radiodiagnosis, All India Institute of Medical Sciences, Bhopal, IND; 4 Cardiology, Lake City Hospital, Bhopal, IND

**Keywords:** cardiovascular risk, rheumatoid arthritis, framingham risk score, cimt, atherosclerosis

## Abstract

Background: Atherosclerosis is the major etiopathogenic factor that decides cardiovascular mortality and morbidity. While inflammation is the putative mechanism for atherosclerosis in various experimental studies, chronic inflammatory state (e.g. in rheumatoid arthritis [RA]) is often neglected as a contributing factor for the development of atherosclerosis. RA patients have two to four times more risk of fatal or non-fatal cardiovascular events, which is not explained by traditional risk factors alone. For example, low-density lipoprotein (LDL) cholesterol levels may not convey the true atherosclerotic risk in RA patients - “the lipid paradox”. Thus, for better risk stratification of future cardiovascular events in RA, the traditional parameters like diabetes, hypertension, and dyslipidemia may not suffice. Newer parameters like carotid intimal-medial thickness (CIMT), coronary calcification scores, and C-reactive protein (CRP) may be needed. This study determined subclinical atherosclerotic load in groups of RA and non-RA patients with comparable Framingham risk scores using CIMT values.

Materials and methods: In this hospital-based cross-sectional study, the RA study group had 64 patients with RA (disease duration > 1 year) and 64 controls were patients with at least one traditional risk factor of cardiovascular disease (e.g., hypertension, cigarette smoking, dyslipidemia, and diabetes mellitus). They were all analyzed for CIMT. The aim was to compare if there was a difference in CIMT scores between groups of RA and non-RA patients, with comparable Framingham score cardiovascular risk categories.

Results: CIMT was significantly higher in the study population compared to controls, indicating increased subclinical atherosclerotic load in the former. Mean CIMT was higher in all age groups in RA patients when compared to the control population (statistically significant in age groups 40-49 years 0.66 ± 0.07 mm vs 0.64 ± 0.06 mm, P < 0.026 and 50-59 years 0.8 ± 0.05 mm vs 0.76 ± 0.05 mm, P < 0.047). CIMT was significantly higher in the intermediate-risk groups (based on the Framingham risk score) in the RA study population when compared with the same risk categories of the control population. Atherogenic indices such as LDL/high-density lipoprotein (HDL) ratio, atherogenic index, and CIMT were significantly higher in the RA patients with more than five years of disease duration than those with a duration of fewer than five years.

Conclusion: Subclinical atherosclerotic load is higher in RA versus controls. The mean CIMT was higher in all age groups in RA compared to the controls. CIMT was significantly higher in the intermediate-risk subgroup (by Framingham risk score) when compared between RA and controls. RA subgroup comparisons based on seropositivity/seronegativity, high/normal CRP, and disease activity (low, intermediate, and high) for CIMT were not found to have statistically significant differences. RA group had lower HDL cholesterol and comparable LDL cholesterol values compared to controls.

## Introduction

Cardiovascular (CV) events are an important cause of mortality worldwide. It has been adequately stressed in the World Health Organisation Global Status Report on Non-communicable Diseases 2010 [1]. Atherosclerosis is the primary etiopathogenic mechanism behind CV events. Although atherosclerosis is recognized as a chronic inflammatory process, chronic systemic inflammatory disorders like rheumatoid arthritis (RA)/ankylosing spondylitis, vasculitis, and connective tissue disorders (e.g. lupus) have not been widely recognized as atherogenic states. These chronic inflammatory disorders have higher cardiovascular risk (CVR) compared to the general population [2,3]. RA patients have excess CV events; some studies depict two to four times the risk of CV mortality and it is even comparable to diabetic patients [4-6].

Management approach to the prevention of CV disorders depends on CVR stratification. Traditional risk factors like diabetes, hypertension, dyslipidemia, smoking, gender, and age have been used to predict CVR in the Framingham risk calculator. Higher-risk (e.g. > 10-year risk of 10% for fatal or non-fatal CV events) individuals are advised pharmacologic interventions like statins and aggressive control of risk factors like hypertension/diabetes while low-risk individuals are advised lifestyle changes alone. However, the above CVR calculators have not been adequately predictive in RA patients [7]. The possible reasons may be the use of non-steroidal anti-inflammatory drugs/steroids and the exclusion of inflammatory markers like C-reactive protein (CRP) and the “Lipid paradox” [8]. “Lipid paradox” is based on the finding that RA patients with low low-density lipoprotein (LDL) cholesterol levels have higher CVR compared to patients with the highest LDL cholesterol, which is in contrast to the general population, where low LDL cholesterol is found to be protective. This is explained by an inflammatory dyslipidemia profile with low LDL cholesterol levels. Thus, there may be a need for other CVR markers like coronary calcium scores, carotid intima-media thickness (CIMT), ankle-brachial index, and CRP for more accurate CVR stratification in RA.

Intima-media thickness is the measure of the combined thickness of the intimal and medial layers of the carotid artery. It is most commonly evaluated by B-mode ultrasound. The increase in CIMT occurs due to hypertrophy of the intimal or medial layers or both. These molecular mechanisms that increase CIMT are similar to the mechanisms responsible for the progression of atherosclerosis [9]. CIMT is the most commonly used surrogate endpoint while coronary artery calcium scoring seems to be the most sensitive for CVR determination. CIMT has the advantage of being non-invasive and has no radiation exposure. Various studies have validated that increasing the CIMT is independently associated with an increased CVR in the general population [10-12]. This has led to the utilization of CIMT as a surrogate marker in RA as well [13].

In this study, we are trying to compare the subclinical atherosclerotic load (using CIMT) in comparable Framingham risk score groups in RA patients vs individuals (without chronic inflammatory disorders). 

## Materials and methods

This observational case-control study was performed at a medical teaching hospital in central India. The study and control groups were enrolled after obtaining Institutional ethical clearance (AIIMS Bhopal Institutional Human Ethics Committee - Post Graduate Research IHECPGRMD010 dated 27th February 2020) and written informed consent from all study participants. All patients (age > 40 years) who attended the medical clinic and/or rheumatology clinic with a diagnosis of RA (ACR/EULAR 2010 Classification Criteria) and disease duration > 1 year were in the RA study group. Controls were patients (age > 40 years) with at least one traditional risk factor of CV disease (such as hypertension, cigarette smoking, dyslipidemia, and diabetes mellitus). All participants were excluded from screening if they had known CV disease/taking primary prophylaxis for CVR (e.g., statins, anti-platelets), structural heart disease, or advanced renal/hepatic failure. In addition, the participants for the control group were excluded if they had known chronic inflammatory diseases such as psoriasis, inflammatory bowel disease, chronic inflammatory arthritis, and lupus.

All study participants were stratified according to Framingham risk scores as mild/intermediate and high CVR at baseline. All participants had CIMT measured as an estimate of subclinical atherosclerosis. CIMT was measured by gray-scale B mode ultrasonography in the common carotid artery bilaterally by examining the common carotid artery up to 2 cm proximal to the bifurcation. The measurement was taken at the site of greatest thickness and three readings were taken from each side at different points within the region of interest. All measurements were taken in diastole, measured in a phase when the lumen diameter was at its smallest and the intima-media thickness was at its largest. The mean value of six readings (three from each side) was decided to be taken as the final CIMT for evaluation.

In addition to relevant biochemical parameters like CRP/erythrocyte sedimentation rate and lipid profile, an echocardiogram/electrocardiogram was also performed. RA group participants had the disease activity score 28 (DAS 28) measured along with other demographic parameters.

Statistical analysis

The sample size was calculated using Gpower 3.1.9.4 version where the mean difference between two independent means (two groups) was applied taking effect size as 0.50, alpha error as 0.05, and power as 80% with allocation ratio as 1. The total sample size was 128 with 64 in each group. Data entry was done using Microsoft Excel and data were processed using SPSS 25 version (IBM Corp, Armonk, NY). Numerical variables were expressed as mean ± SD if normally distributed and median and interquartile for non-normal distribution. Categorical variables were summarized as number percentages. Association between categorical variables was analyzed using the chi-square test and the correlation between continuous variables using Pearson's/Spearman's based on normality of distribution. The odds ratio was calculated. The mean difference between the two groups was analyzed using an unpaired t-test/Wilson signed rank test or Mann-Whitney U test based on the normality. Analysis of variance (ANOVA) was applied to test the significance when there are more than two groups followed by post-hoc tests. A P-value <0.05 was considered significant.

## Results

Sixty-four participants in the RA study group and 64 in the control group were enrolled in the study and were compared for CIMT scores. The mean age of the RA study population was 46.7 years and that of the control population was 44.1 years. The majority of the RA study population was in the age group 40-49 years (68.8%). The mean duration of RA disease in the study was 5.1 ± 3.5 years, with 51% of patients having a duration of illness of fewer than five years. The majority of the patients were seropositive (76.5%) and most were already taking treatment at the time of enrolment. Seropositive is defined as either rheumatoid factor or anti-cyclic citrullinated peptide positivity. Most of the patients in the study population had an intermediate risk when categorized according to Framingham risk score distribution (37.5%). A comparison of the Framingham risk scores in the study and control groups is listed in Table 1.

**Table 1:** Comparison of the Framingham risk score in the study and control groups HDL, high-density lipoprotein; BP, blood pressure; CIMT, carotid intima-media thickness.

Components		Study Population	Control Population	P-value
Age		46.7	44.1	0.627
Sex	Male	14 (21.8)	13 (20.3)	0.654
	Female	50 (78.1)	51 (79.6)	0.644
Total cholesterol (mg/dL)		184.9 (150-204)	171.9 (148-192)	0.078
Smoking		6 (9.3%)	8 (12.5%)	0.124
HDL (mg/dL)		37.6 (30-45)	46.1 (39-53)	0.001
Systolic BP (mm Hg)		116 (104-130)	114 (103-125)	0.81
CIMT		0.74 ± 0.08	0.72 ± 0.07	0.061

Mean CIMT was higher in all age groups of the RA patients when compared to the similar age groups of the control population (statistically significant in age groups 40-49 years 0.66 ± 0.07 mm vs 0.64 ± 0.06 mm; P < 0.026 and 50-59 years 0.8 ± 0.05 mm vs 0.76 ± 0.05 mm; P < 0.047). CIMT was significantly higher in the intermediate-risk Framingham risk score in the RA study population when compared with the same risk categories of the control population (Table 2). There was no significant difference noted in LDL/HDL (high-density lipoprotein), CIMT, duration of illness, DAS score, and Framingham risk scores when compared in the seropositive and seronegative groups of the RA study population. There was no significant difference noted in Framingham risk scores and CIMT values when the CRP-positive subpopulation was compared with the CRP-negative subpopulation. 

**Table 2:** Comparison of Framingham risk score categories and CIMT CIMT, carotid intima-media thickness.

Framingham Risk Score	Study Population		Control Population		P-value
	Number	CIMT (Mean)	Number	CIMT (Mean)	
Low	20	0.64	22	0.60	0.06
Intermediate	24	0.78	20	0.72	0.005
High	20	0.86	22	0.90	0.065

The mean DAS28 CRP score was 4.68 ± 0.73. The majority of the population had moderate disease activity (53%) while 34.3% of patients had high disease activity. There was no statistically significant difference noted in CIMT and biochemical parameters among the three subpopulations of the DAS28 score (namely low, moderate, and high activity) (Table 3). Patients in the RA study population had statistically higher levels of triglycerides (TGs), higher levels of non-HDL cholesterol, and lower levels of HDL cholesterol. There was no significant difference noted in the values of LDL cholesterol between the study and control population. The atherogenic dyslipidemia indices like LDL/HDL ratio and atherogenic index were higher in the RA study population when compared to the controls.

**Table 3:** Comparison of DAS 28 CRP score, LDL/HDL, and CIMT DAS 28, disease activity score 28; CRP, C-reactive protein; LDL, low-density lipoprotein; HDL, high-density lipoprotein; CIMT, carotid intima-media thickness.

DAS 28 CRP Score		LDL/HDL	CIMT
<3.2	Mean	3.44	0.74
	SD	0.32	0.07
>3.2-5.1	Mean	2.60	0.77
	SD	0.41	0.08
>5.1	Mean	3.52	0.78
	SD	0.48	0.07
P-value		0.39	0.18

## Discussion

This study aimed to explore the subclinical atherosclerotic load in RA patients. The CIMT and biochemical parameters in RA were compared with those in the controls. We wanted to ascertain whether RA patients had more risk of developing CV disease when compared with patients who had a traditional risk factor. We used Framingham risk scores to stratify each study group into CVR risk categories and then compared each risk subgroup for CIMT values.

Multiple studies have focussed on the increased CVR in RA. The CARRE (CARdiovascular research and RhEumatoid arthritis) study demonstrated that RA imparts a similar risk of developing CV events as with diabetes mellitus [4]. A cohort study demonstrated that RA patients were at four times more risk of developing CV events than the general population [14]. Multiple studies have hypothesized the risk to be elevated given a sustained inflammatory state in these patients. This was attributed to elevated levels of tumor necrosis factor (TNF)-alpha and other inflammatory mediators [15-18]. Studies attribute the changes seen in lipoprotein composition in RA secondary to the above inflammatory mediators [19]. Elevated CRP levels are associated with insulin resistance, which could be a possible explanation for the worsening of dyslipidemia seen in RA [20]. 

Dyslipidemia in RA has a different pattern as compared to traditional metabolic syndrome. Patients with active RA tend to have lower values of total cholesterol (TC), LDL, and HDL levels [21-24]. In our study, patients in the RA study group had statistically higher TG levels, higher levels of non-HDL cholesterol, and lower HDL levels. The atherogenic dyslipidemia indices like non-HDL cholesterol, LDL/HDL ratio, and atherogenic index were higher in the RA study population. The LDL/HDL ratio was significant, most likely because of reduced HDL levels seen in the study population. A study was done at AIIMS New Delhi that demonstrated lower levels of HDL and increased levels of non-HDL cholesterol in RA [25]. In our study, there was no significant difference noted in the values of LDL between the study and control population. Multiple studies have tried to evaluate the reasons for decreased levels of cholesterol and the "lipid paradox" in RA. The hypothesis suggested includes circulating autoantibodies, immune complex deposition, increased oxidation/clearance, and reduced synthesis [26,27].

CIMT has been explored in RA in several studies. In our study, CIMT was significantly higher in the RA study group. The upper limit of CIMT was taken as 0.72 mm for our study. Mean CIMT was higher in all age groups in RA patients when compared to the control group (statistically significant in the age group 40-49 years and 50-59 years). A landmark study done in the Indian setting in RA patients demonstrated that age >42 years, disease duration >6 years, and tender joint count >5 predicted an increased risk of having abnormal CIMT [28]. Multiple studies have validated an increased CIMT in patients of RA when compared with healthy controls [29]. In our study, CIMT was found to be higher and had statistical significance in the intermediate-risk score categories of Framingham risk score when compared with the control population. In a meta-analysis by Ferket et al. (2014), different modalities in addition to the Framingham risk score to improve CVR assessment were compared. They evaluated CT coronary calcium score, CIMT, high-sensitivity CRP, and ankle-brachial index and found that CT coronary calcium scoring performed the best to better stratify those at risk of worse CV outcomes. They suggested that the risk stratification is most compelling when CT coronary calcium scoring is added to the intermediate-risk population [30]. Thus, the association between the Framingham risk score and additional CV risk factors, exposure to corticosteroids, and RA disease duration must be explored further as early statin/anti-platelet use might help in reducing subsequent CV morbidity and mortality in RA patients. Efforts must be made to improve the prediction of risk by combining the Framingham risk score with CIMT and coronary calcium score to derive better risk stratification.

The major limitation of this study was the low sample size to statistically compare the Framingham risk categories with CIMT. Most patients in the study were already taking corticosteroids and disease-modifying antirheumatic drugs (DMARDs), all affecting the lipoprotein parameters as well as CIMT. The strength of the study is a comparison of RA with traditional CVR factors for atherosclerosis, using the non-invasive parameter (CIMT).

## Conclusions

This study aimed to explore the subclinical atherosclerotic load in RA. CIMT was significantly higher in the study population indicating increased subclinical atherosclerotic load and the mean CIMT was higher in all age groups in RA compared to the controls. CIMT was significantly higher in the intermediate-risk subgroup (by Framingham risk score) when compared between RA and controls. RA subgroup comparisons, based on seropositivity/seronegativity, high/normal CRP, and disease activity (low, intermediate, high) for CIMT values, were not found to have statistically significant differences. An interesting finding was that HDL values were significantly lower and LDL/HDL ratio was significantly higher in RA patients who were CRP-positive. RA group had lower HDL cholesterol and comparable LDL cholesterol values compared to controls. However, atherogenic dyslipidemia indices like LDL/HDL ratio and atherogenic index were higher in the RA group, especially with the disease duration >5 years. The lower LDL cholesterol levels in RA may give a false complacency concerning CVR estimation and may need to be replaced by other parameters like the atherogenic index. For an accurate estimate of CVR in RA, the association between the inflammatory lipid profile, exposure to corticosteroids, and RA disease duration must be explored further in prospective cohort studies. Such studies may guide early interventions in the form of lifestyle changes and anti-platelets/statin use in reducing CVR. Efforts must be made to improve the prediction of CVR by combining the Framingham risk score with CIMT, newer lipoprotein parameters, and coronary calcium score, in chronic inflammatory disorders.
